# Effectiveness and Safety of Imatinib and Nilotinib in Chronic Myeloid Leukemia: A Single-Center Study From Southern Morocco

**DOI:** 10.7759/cureus.113439

**Published:** 2026-07-27

**Authors:** Mossaab El Mousadik, Houda El Khayat, Layla El Hizazi, Oumaima Bounar, Omar Halloumi, Rida Fatima Ezzahra, Imad Chakri, Afaf Bouqoufi, Laila Lahlou, Salma Fares

**Affiliations:** 1 Hematology, Faculty of Medicine and Pharmacy of Agadir, Ibn Zohr University, Agadir, MAR; 2 Epidemiology and Clinical Research Laboratory, Faculty of Medicine and Pharmacy of Agadir, Ibn Zohr University, Agadir, MAR; 3 Medical and Clinical Pharmacology, Faculty of Medicine and Pharmacy of Agadir, Ibn Zohr University, Agadir, MAR

**Keywords:** adverse events, chronic myeloid leukemia, imatinib, nilotinib, tyrosine kinase inhibitors

## Abstract

Introduction: The prognosis of chronic myeloid leukemia (CML) has been revolutionized following the advent of tyrosine kinase inhibitors (TKIs); however, access to molecular monitoring, resistance mutation screening, and second- and third-generation TKIs remains limited in resource-constrained countries. The aim of this study was to describe the efficacy and safety profile of imatinib and nilotinib in southern Morocco.

Methods: This was a single-center, retrospective study conducted in the Clinical Hematology Department of the Mohammed VI University Hospital of Agadir over a ten-year period (2014-2024), including 80 patients with CML in the chronic or accelerated phase at diagnosis. All patients received first-line imatinib, with a switch to nilotinib in cases of resistance, relapse, or intolerance. Molecular response was assessed by quantitative reverse transcription polymerase chain reaction (RT-PCR) according to the International Scale (IS), with a favorable response defined as a BCR::ABL1 transcript of ≤ 1% IS at 12 months. Survival was estimated using the Kaplan-Meier method.

Results: Among the 80 patients, the mean age was 47.0 ± 17.3 years, with a slight female predominance (M/F sex ratio = 0.9). The Sokal score classified 42.5% of patients as high risk. Under imatinib, a favorable molecular response at 12 months was achieved in 50% of patients, with primary resistance in the other half. As second-line therapy, nilotinib achieved a favorable response in 32.3% of the 31 treated patients. Grade ≥ 3 toxicity was observed in 12.5% of patients on imatinib and 19.4% on nilotinib, with no grade ≥ 3 cardiovascular events. After a median follow-up of 48 months, overall survival (OS) remained at 89.9% beyond 60 months, whereas event-free survival (EFS) under imatinib was shorter (median 24 months). The Sokal score was significantly associated with EFS (p = 0.007).

Conclusions: Our series is notable for the young age of patients, a female predominance, and a high proportion of high-risk Sokal scores; a profile consistent with other Moroccan cohorts. Despite a high rate of imatinib resistance, likely related to the unfavorable prognostic profile, diagnostic delay, and limitations in molecular monitoring, OS remained excellent. These findings highlight the value of expanded access to second-generation TKIs as first-line therapy and to resistance mutation screening in our setting.

## Introduction

Chronic myeloid leukemia (CML) is a myeloproliferative neoplasm characterized by the reciprocal balanced translocation t(9;22)(q34;q11.2), which generates the BCR::ABL1 fusion gene and results in constitutive tyrosine kinase activity, leading to uncontrolled proliferation of myeloid cells with preserved maturation [1]. The introduction of tyrosine kinase inhibitors (TKIs) has dramatically changed the natural history of CML, with reported 10-year overall survival (OS) rates exceeding 80% [2,3]. Despite these advances, resistance to first-line TKIs occurs in approximately 15%-20% of patients and may increase to as high as 50% after multiple lines of therapy [4].

The European LeukemiaNet (ELN) currently recommends BCR::ABL1 kinase domain mutation analysis in patients with suspected (warning) or confirmed treatment failure during molecular monitoring, as mutation profiles are essential for selecting the most appropriate subsequent TKI [5]. In resource-limited countries, including Morocco, access to molecular monitoring, resistance mutation testing, and second- and third-generation TKIs remains limited, as reported in the few published Moroccan studies [6,7].

This study aimed to evaluate the effectiveness and safety of first-line imatinib and second-line nilotinib by assessing molecular response, survival outcomes, and treatment-related adverse events in patients with CML treated at a tertiary referral center in a resource-limited region of southern Morocco.

## Materials and methods

Study design and patient population

This retrospective, descriptive, analytical, single-center study was conducted at the Department of Clinical Hematology of Mohammed VI University Hospital in Agadir, the main hematology referral center for southern Morocco. The study included patients diagnosed with chronic- or accelerated-phase CML between January 2014 and December 2024.

The diagnosis of CML was confirmed by the presence of the Philadelphia chromosome and/or the BCR::ABL1 fusion transcript. Patients with incomplete medical records, those without molecular monitoring during TKI therapy, and those diagnosed with blast-phase CML were excluded. 

Treatment and response assessment

All patients (n = 80) received first-line imatinib at a daily dose of either 400 mg or 600 mg. Patients who developed resistance, intolerance, or relapse subsequently received second-line nilotinib at a daily dose of either 600 mg or 800 mg. Treatment doses were determined according to the treating physician's clinical judgment and institutional practice. Molecular response was assessed using quantitative reverse transcription polymerase chain reaction (RT-PCR) for BCR::ABL1 transcripts and reported according to the International Scale (IS) [8]. Treatment response milestones were interpreted according to the 2025 ELN recommendations [5].

Because of the retrospective design and disparities in access to molecular monitoring in our region, a favorable response was defined as a BCR::ABL1 transcript level ≤1% (IS) at 12 months, corresponding to the molecular equivalent of a complete cytogenetic response (CCyR). Resistance was defined as failure to achieve a favorable response (BCR::ABL1 >1% IS at 12 months) and/or a BCR::ABL1 transcript level >10% IS at six months when this assessment was available. Relapse was defined as the loss of a previously achieved CCyR, documented by an increase in the BCR::ABL1 transcript level to >1% IS. Treatment-related adverse events were graded according to the Common Terminology Criteria for Adverse Events (CTCAE), version 5.0 [9].

Statistical analysis

Statistical analyses were performed using Jamovi software (version 2.6.26, https://www.jamovi.org). Categorical variables are presented as frequencies and percentages. The distribution of continuous variables was assessed using Q-Q plots and the Shapiro-Wilk test. Normally distributed variables are reported as mean ± standard deviation, whereas non-normally distributed variables are presented as median and interquartile range (IQR).

OS and event-free survival (EFS) were estimated using the Kaplan-Meier method with 95% confidence intervals (CI). OS was defined as the time from diagnosis to death from any cause or the date of the last follow-up (censored observations). EFS was defined as the time from initiation of imatinib therapy to the first occurrence of primary imatinib resistance, relapse after an initial response, progression to blast phase, or death. EFS was compared across Sokal risk groups using the log-rank test [10]. A two-sided p-value <0.05 was considered statistically significant.

## Results

Baseline characteristics

**Figure 1:**
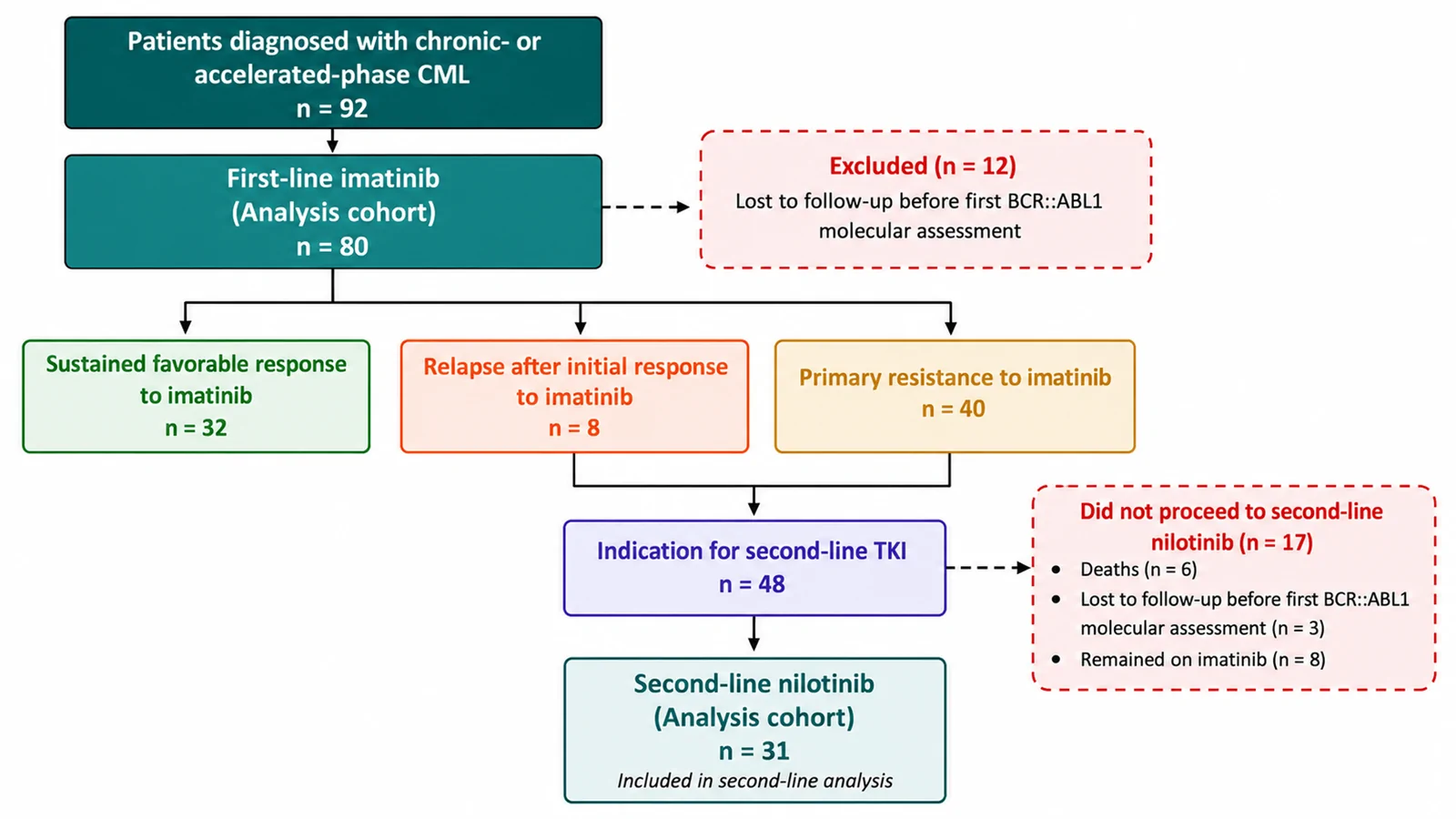
Flow diagram CML: chronic myeloid leukemia; TKI: tyrosine kinase inhibitor

A total of 80 patients met the eligibility criteria and were included in the final analysis (Figure 1).

The mean age was 47.0 ± 17.3 years (range, 13-91 years), with a slight female predominance (male-to-female ratio, 0.9). Thirteen patients (16.3%) had at least one comorbidity, most commonly hypertension (6.3%). At diagnosis, 78 patients (97.5%) were in the chronic phase, and two (2.5%) were in the accelerated phase. According to the Sokal score, 34 patients (42.5%) were classified as high risk, 27 (33.8%) as low risk, and 19 (23.8%) as intermediate risk (Table 1).

**Table 1:** Baseline demographic, clinical, laboratory, and prognostic characteristics of the study population (n = 80) *values are presented as mean ± standard deviation; **values are presented as counts and percentages (%); ***values are presented as median (interquartile range, IQR).

Characteristic	Value (n = 80)	Reference range
Demographic characteristics		
Age, years*	47.0 ± 17.3	—
Female sex**	42 (52.5)	—
Male sex**	38 (47.5)	—
Male-to-female ratio	0.9	—
Any comorbidity**	13 (16.3)	—
─ Hypertension**	5 (6.3)	—
─ Heart disease**	3 (3.8)	—
─ Diabetes mellitus**	3 (3.8)	—
─ Chronic kidney disease**	2 (2.5)	—
Clinical and laboratory characteristics at diagnosis		
Chronic phase**	78 (97.5)	—
Accelerated phase**	2 (2.5)	—
Spleen size below the left costal margin, cm***	5.0 (0.0–14.3)	Not palpable
Hemoglobin, g/dL***	10.0 (9.0–10.0)	Female: 12.0–16.0; Male: 13.0–16.5
Platelet count, ×10^3^/µL***	398 (251–603)	150–400
Neutrophil count, ×10^3^/µL***	70.0 (39.8–130.2)	1.5–7.5
Peripheral blood blasts, %***	2.0 (1.0–4.0)	0
Basophil count, /µL***	3,380 (64–7,900)	0–100
Myelemia, %*	41.1 ± 17.0	0
Prognostic stratification		
Low Sokal risk**	27 (33.8)	—
Intermediate Sokal risk**	19 (23.8)	—
High Sokal risk**	34 (42.5)	—

Response to first-line imatinib

All patients (n = 80) received first-line imatinib. The daily dose was 400 mg in 73 patients (91.3%) and 600 mg in seven patients (8.8%). The median duration of treatment was 20.0 months (IQR, 6.0-51.5 months). A favorable molecular response at 12 months (BCR::ABL1 ≤1% IS) was achieved in 40 patients (50.0%), whereas the remaining 40 patients (50.0%) met the criteria for primary resistance. At the end of follow-up, 32 patients (40.0%) maintained a favorable response, 40 (50.0%) had persistent resistance, and eight (10.0%) experienced relapse after an initial response. Among responders, the median duration of response was 46.0 months (IQR, 34.8-65.5 months) (Table 2).

**Table 2:** Treatment characteristics and molecular response to first-line imatinib (n = 80) *values are presented as median (interquartile range, IQR); **values are presented as counts and percentages (%); † favorable response was defined as a BCR::ABL1 transcript level ≤1% on the International Scale (IS) at 12 months. This category includes patients who achieved complete cytogenetic response (CCyR), major molecular response (MMR), or deep molecular response (DMR).

Characteristic	Value (n= 80)
Treatment	
Imatinib 400 mg/day**	73 (91.3)
Imatinib 600 mg/day**	7 (8.8)
Best molecular response at 12 months	
Deep molecular response (DMR)**	31 (38.7)
Major molecular response (MMR)**	7 (8.8)
Complete cytogenetic response (CCyR)**	2 (2.5)
Primary resistance**	40 (50.0)
Favorable response†**	40 (50.0)
Status at last follow-up	
Sustained favorable response**	32 (40.0)
Persistent resistance**	40 (50.0)
Relapse after initial response**	8 (10.0)
Treatment duration (months)*	20.0 (6.0–51.5)
Duration of response among responders (n = 40), months*	46.0 (34.8–65.5)

Response to second-line nilotinib

Among the 47 patients with an indication for second-line TKI therapy, 31 received nilotinib and were included in the second-line analysis (Figure 1). Of these, 27 (87.1%) had switched because of primary resistance to imatinib and four (12.9%) because of relapse after an initial response. Nilotinib was administered at a daily dose of 600 mg in 12 patients (38.7%) and 800 mg in 19 patients (61.3%). The median treatment duration was 6.0 months (IQR, 6.0-18.5 months). A favorable molecular response at 12 months was achieved in 10 patients (32.3%), whereas 21 (67.7%) were classified as resistant. At the end of follow-up, eight patients (25.8%) maintained a favorable response, 21 (67.7%) had persistent resistance, and two (6.5%) experienced relapse. Among responders, the mean duration of response was 17.9 ± 9.0 months (Table 3).

**Table 3:** Treatment characteristics and molecular response to second-line nilotinib (n = 31) *values are presented as median (interquartile range, IQR); **values are presented as counts and percentages (%); ‡ values are presented as mean ± standard deviation; † favorable response was defined as a BCR::ABL1 transcript level ≤1% on the International Scale (IS) at 12 months. This category includes patients who achieved complete cytogenetic response (CCyR), major molecular response (MMR), or deep molecular response (DMR).

Characteristic	Value (n = 31)
Treatment	
Nilotinib 600 mg/day**	12 (38.7)
Nilotinib 800 mg/day**	19 (61.3)
Best molecular response at 12 months	
Deep molecular response (DMR)**	7 (22.5)
Major molecular response (MMR)**	2 (6.5)
Complete cytogenetic response (CCyR)**	1 (3.2)
TKI resistance**	21 (67.7)
Favorable response†**	10 (32.3)
Status at last follow-up	
Sustained favorable response**	8 (25.8)
Persistent resistance**	21 (67.7)
Relapse after initial response**	2 (6.5)
Treatment duration (months)*	6.0 (6.0–18.5)
Duration of response among responders (n = 10), months‡	17.9 ± 9.0

Safety of imatinib

Grade ≥3 hematologic adverse events occurred in 10 patients (12.5%), with anemia being the most frequent event (6.3%). No grade ≥3 non-hematologic adverse events were observed (Table 4).

**Table 4:** Adverse events associated with first-line imatinib according to severity (n = 80) *values are presented as counts and percentages (%).

Adverse event	Total*	Grade <3*	Grade ≥3*
Hematologic adverse events			
Anemia	30 (37.5)	25 (31.3)	5 (6.3)
Thrombocytopenia	14 (17.5)	11 (13.8)	3 (3.8)
Neutropenia	5 (6.3)	3 (3.8)	2 (2.5)
Non-hematologic adverse events			
Vomiting	8 (10.0)	8 (10.0)	0
Fluid retention/edema	7 (8.8)	7 (8.8)	0
Pruritus	5 (6.3)	5 (6.3)	0
Epigastric pain	5 (6.3)	5 (6.3)	0
Headache	4 (5.0)	4 (5.0)	0
Skin rash	3 (3.8)	3 (3.8)	0
Fatigue	3 (3.8)	3 (3.8)	0
Diarrhea	2 (2.5)	2 (2.5)	0
Muscle cramps	2 (2.5)	2 (2.5)	0
Elevated transaminases	2 (2.5)	2 (2.5)	0
Hyperglycemia	2 (2.5)	2 (2.5)	0
Abdominal pain	1 (1.3)	1 (1.3)	0
Hypertension	1 (1.3)	1 (1.3)	0

Safety of nilotinib

Grade ≥3 hematologic adverse events occurred in six patients (19.4%), predominantly thrombocytopenia (16.1%). Only one patient experienced a grade ≥3 non-hematologic adverse event (skin rash). No grade ≥3 cardiovascular adverse events were observed (Table 5).

**Table 5:** Adverse events associated with second-line nilotinib according to severity (n = 31) *values are presented as counts and percentages (%).

Adverse event	Total*	Grade <3*	Grade ≥3*
Hematologic adverse events			
Thrombocytopenia	9 (29.0)	4 (12.9)	5 (16.1)
Neutropenia	5 (16.1)	5 (16.1)	0
Anemia	4 (12.9)	3 (9.7)	1 (3.2)
Non-hematologic adverse events			
Skin rash	4 (12.9)	3 (9.7)	1 (3.2)
Elevated transaminases	2 (6.5)	2 (6.5)	0
Muscle cramps	1 (3.2)	1 (3.2)	0
Fatigue	1 (3.2)	1 (3.2)	0
Dyslipidemia	1 (3.2)	1 (3.2)	0
QT interval prolongation	1 (3.2)	1 (3.2)	0
Abdominal pain	1 (3.2)	1 (3.2)	0

Survival outcomes

**Figure 2:**
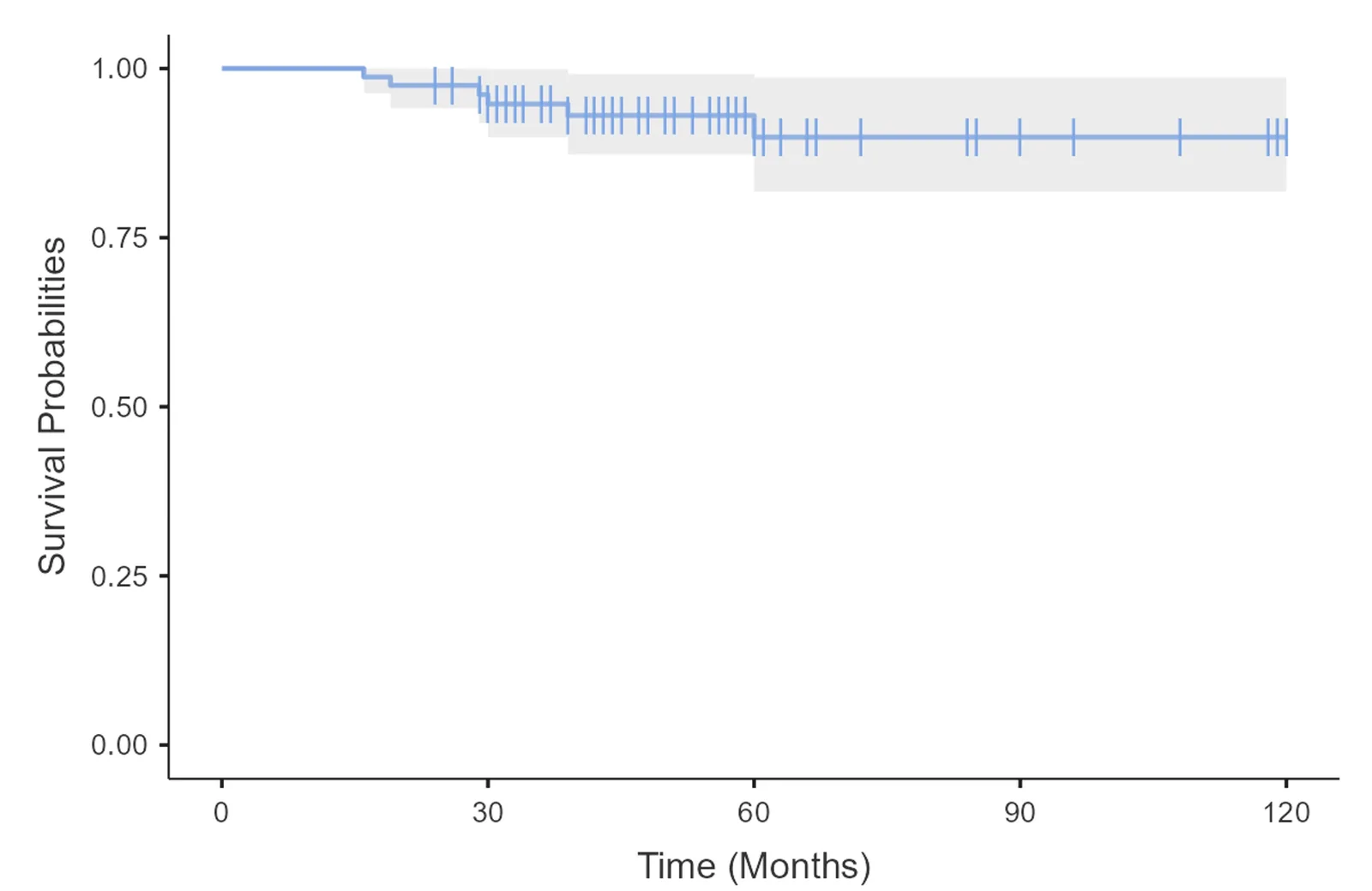
Kaplan-Meier overall survival (OS) curve

After a median follow-up of 48.0 months (IQR, 34-72 months), six deaths were recorded. The median OS was not reached, and the estimated five-year OS was 89.9% (95% CI, 77.3%-95.6%) (Figure 2).

**Figure 3:**
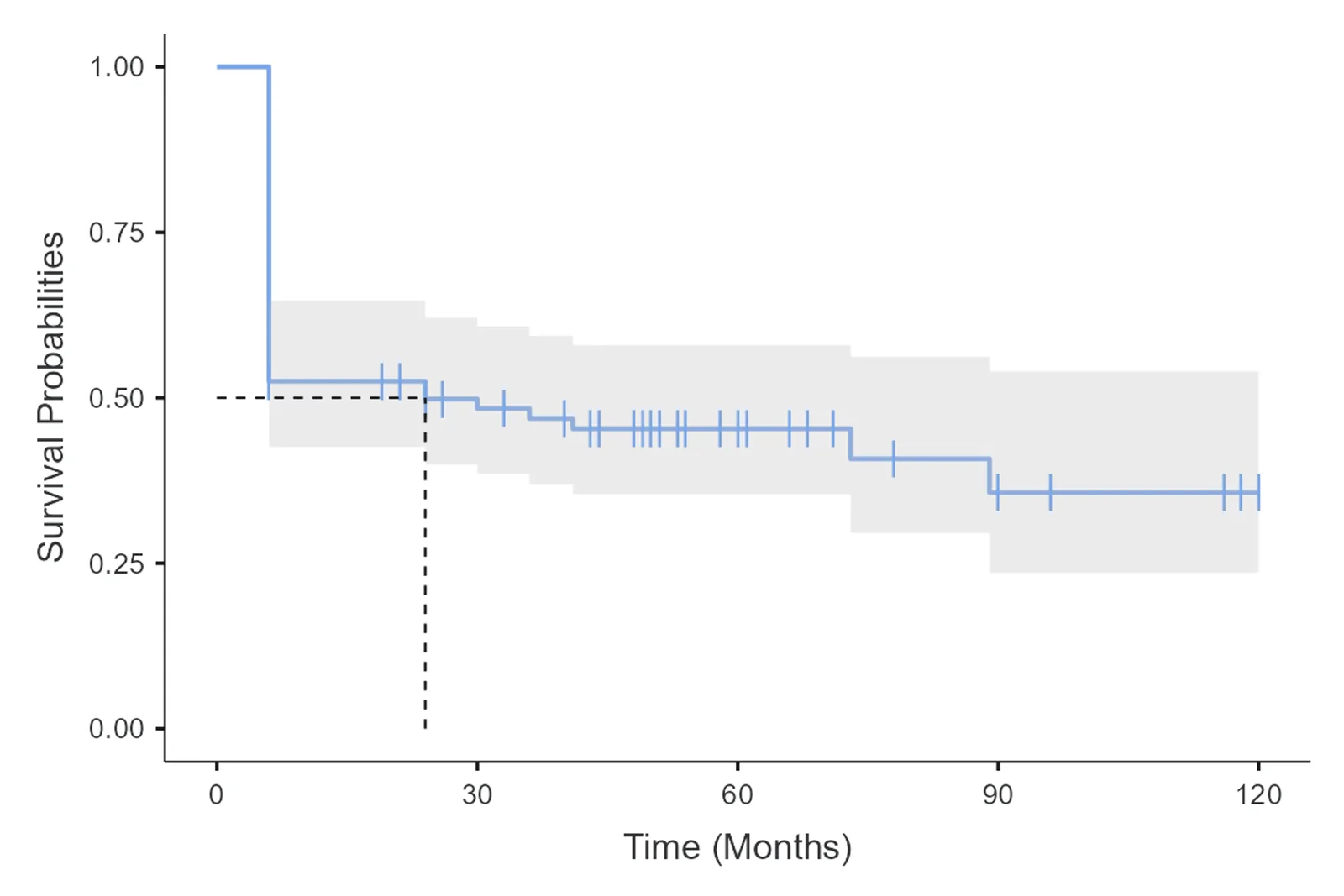
Kaplan-Meier event-free survival (EFS) with first-line imatinib

The median EFS with first-line imatinib was 24 months (Figure 3).

**Figure 4:**
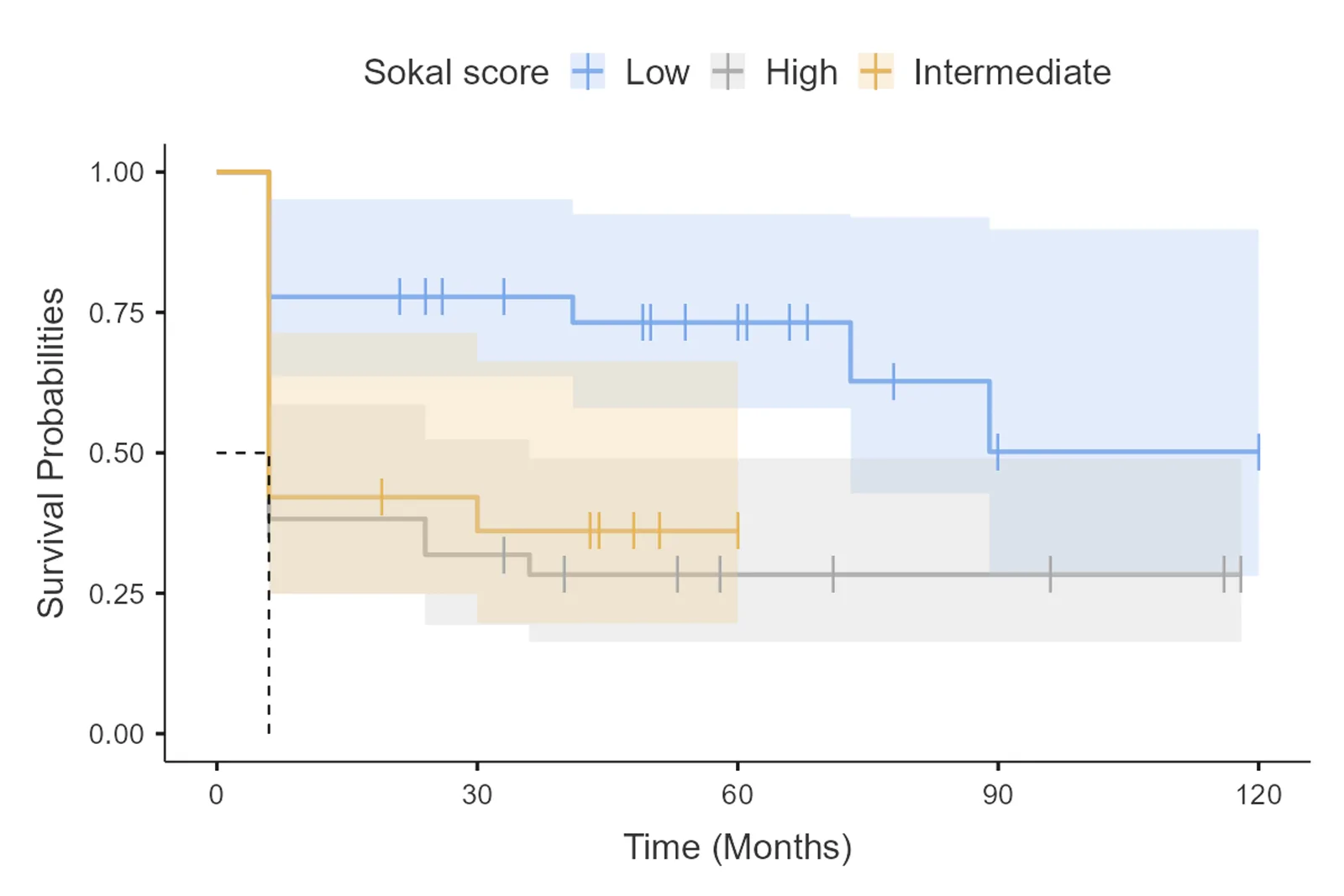
Kaplan-Meier event-free survival (EFS) according to Sokal risk group

The EFS differed significantly according to the Sokal risk group (log-rank p = 0.007). Patients in the low-risk group had a prolonged EFS, with the median not reached, whereas those in the intermediate- and high-risk groups had a median EFS of six months. Pairwise comparisons confirmed significantly longer EFS in the low-risk group than in both the high-risk (p = 0.002) and intermediate-risk (p = 0.011) groups, whereas no significant difference was observed between the intermediate- and high-risk groups (p = 0.605) (Figure 4).

## Discussion

This study describes the effectiveness and safety of imatinib and nilotinib in a real-world cohort of 80 patients with CML treated over a 10-year period at Mohammed VI University Hospital in Agadir, the main hematology referral center in southern Morocco.

The mean age of our patients was 47.0 ± 17.3 years, which is substantially lower than the median age of 57-60 years reported at diagnosis in European CML registries [11]. This relatively young age is consistent with findings from the Moroccan cohort reported by the Casablanca group, where the median age at diagnosis was 41.5 years [6]. We also observed a slight female predominance (male-to-female ratio, 0.9), whereas CML is generally more common in men, with male-to-female ratios ranging from 1.2 to 1.7 in European registries [11]. Similar findings have been reported in Moroccan cohorts, including the Casablanca study (male-to-female ratio, 0.7) [6] and a nationwide series of 826 patients diagnosed between 1992 and 2023, which reported a median age of 45 years and an almost equal sex distribution (male-to-female ratio, 1.03) [7]. These observations suggest that the epidemiological profile of CML in Morocco may differ from that reported in Western populations.

Regarding prognostic stratification, 42.5% of our patients were classified as high risk according to the Sokal score, compared with 33.8% classified as low risk. This predominance of high-risk disease is consistent with the Casablanca cohort, where 56% of patients were classified as high risk, a finding attributed to delayed diagnosis [6]. The 2025 ELN recommendations suggest that younger patients and those with high-risk prognostic scores, particularly according to the EUTOS Long-Term Survival (ELTS) score, may benefit from second-generation TKIs as first-line therapy [5]. Given the relatively young age of our cohort and the high proportion of patients with high-risk disease, earlier use of second-generation TKIs may represent an appropriate therapeutic strategy in our setting.

Only 50% of patients achieved a favorable molecular response (BCR::ABL1 ≤1% on the IS, corresponding to the molecular equivalent of a CCyR] after 12 months of first-line imatinib therapy, whereas the remaining patients met the criteria for primary resistance. This response rate is comparable to that reported by the Casablanca group, where the estimated 12-month CCyR rate was 43% [6], but substantially lower than the 81% CCyR rate reported in the IRIS trial [12]. In our cohort, EFS differed significantly according to Sokal risk group (log-rank p = 0.007), with patients in the low-risk group experiencing significantly longer EFS than those in the intermediate- and high-risk groups. These findings suggest that baseline disease risk may have contributed to the poorer treatment outcomes observed in our cohort.

Among patients receiving second-line nilotinib, a favorable molecular response at 12 months was achieved in 32.3%, whereas 67.7% met the criteria for resistance. In contrast, the ENESTnd (Evaluating Nilotinib Efficacy and Safety in Clinical Trials-Newly Diagnosed Patients) trial reported a 10-year major molecular response rate of 77.7% with first-line nilotinib [13], and a real-world study comparing second-line dasatinib and nilotinib reported a 12-month major molecular response rate of 76.9% in the nilotinib group [14]. These differences suggest a high burden of treatment resistance in our population and highlight the importance of performing BCR::ABL1 kinase domain mutation analysis to guide subsequent TKI selection.

Regarding safety, grade ≥3 hematologic adverse events occurred in 12.5% of patients treated with imatinib, whereas no grade ≥3 non-hematologic adverse events were observed. In the IRIS (International Randomized Study of Interferon and STI571) trial, severe non-hematologic toxicities were more frequently reported [12]. Among patients treated with nilotinib, grade ≥3 hematologic adverse events occurred in 19.4%, predominantly thrombocytopenia (16.1%), whereas only one patient developed a grade ≥3 non-hematologic adverse event (skin rash). No grade ≥3 cardiovascular adverse events were observed. In the 10-year ENESTnd analysis, cumulative cardiovascular event rates reached 23.5% in patients receiving nilotinib 800 mg/day, compared with 3.6% in those receiving imatinib [13]. The relatively short duration of nilotinib exposure in our cohort (median, six months), together with the younger age of our patients, likely explains the absence of severe cardiovascular complications.

Despite the relatively high rates of treatment resistance observed in our study, OS remained favorable, with an estimated five-year OS of 89.9%. This finding is consistent with the long-term outcomes reported in the IRIS trial, which demonstrated a 10-year OS of 83.3% [2], the ENESTnd study, which reported a 10-year OS of 88.3% in the imatinib arm [13], and the Casablanca cohort, which reported a 10-year OS of 86% [6]. Similar observations have recently been reported in other cohorts. In one study including 131 patients, CML-specific survival exceeded 90% at 10 years among patients with BCR::ABL1 transcript levels between 0.1% and 1% or between 1% and 10% after two years of treatment [15]. Likewise, the German CML-IV study, which included 1,342 patients, reported an approximately 80% 10-year survival rate among patients with molecular treatment failure (BCR::ABL1 >1% at 12 months), whereas survival decreased to approximately 55% when transcript levels remained >10% at 12 months [16]. Although these findings require further confirmation, they support the more individualized therapeutic approach proposed by the 2025 ELN recommendations, particularly in older patients or those with significant comorbidities for whom switching TKIs may not be feasible because of contraindications or intolerance [5].

This study has several limitations. Its retrospective, single-center design and relatively small sample size limit the generalizability of the findings. In addition, medication adherence was not systematically assessed, precluding evaluation of its potential impact on treatment response. The exclusion of patients without molecular monitoring may also have introduced selection bias, as it preferentially included patients with better access to healthcare and follow-up. Furthermore, the absence of systematic BCR::ABL1 mutation analysis, together with disparities in access to molecular monitoring, prevented characterization of resistance mechanisms and precluded strict application of the ELN response milestones. Finally, the short duration of nilotinib exposure restricted the assessment of its long-term efficacy and safety, particularly with regard to cardiovascular toxicity. Despite these limitations, this study provides real-world evidence on the effectiveness and safety of imatinib and nilotinib in a region where published data remain scarce, and it may help inform the management of CML in resource-limited settings.

## Conclusions

In this single-center study from southern Morocco, CML was characterized by a relatively young age at diagnosis, a slight female predominance, and a high proportion of patients with high-risk Sokal scores. First-line imatinib was associated with a high rate of primary resistance, and switching to nilotinib resulted in only modest molecular responses in a setting where BCR::ABL1 mutation testing was unavailable. Despite these findings, OS remained favorable, highlighting the dissociation between relatively short EFS and prolonged OS when subsequent therapeutic options remain available. These results support expanding access to molecular monitoring, BCR::ABL1 mutation analysis, and first-line second-generation TKIs, particularly for younger patients and those with high-risk disease, to optimize the management of CML in resource-limited settings.
